# The effect of reticulocyte hemoglobin content on the diagnosis of iron deficiency anemia: A meta-analysis study

**DOI:** 10.5937/jomb0-31435

**Published:** 2022-02-02

**Authors:** Merve Kılıç, Aysel Özpınar, Mustafa Serteser, Meltem Kilercik, Muhittin Serdar

**Affiliations:** 1 Acıbadem Mehmet Ali Aydinlar University, School of Medicine, Dept. Medical Biochemistry, Istanbul, Turkey; 2 Dogus University, School of Vocational, Dept. Anesthesia Program, Istanbul, Turkey

**Keywords:** Reticulocyte Hemoglobin Content, Iron Deficiency Anemia, Transferrin saturation, Mean Corpus Volume, Ferritin, Meta-Analysis, Sadržaj retikulocitnog hemoglobina, anemija usled nedostatka gvožđa, zasićenje transferina, srednja zapremina tela, feritin, meta-analiza

## Abstract

**Background:**

Iron deficiency anemia (IDA) is the most common type of anemia worldwide and has many adverse effects on life quality. This meta-analysis study aims to show that reticulocyte hemoglobin content (CHr) is more effective than routinely used parameters in the diagnosis of IDA.

**Methods:**

Comprehensive and systematic research was done using international databases including PubMed, Web of Science, Cochrane Library, Science Direct, and Google Scholar, which contain all articles published on IDA until December 29, 2020. Seventeen articles were included in the meta-analysis.

**Results:**

The analyses found the Cohen's deffect size (Standardized Mean Difference) values of the parameters. Accordingly, CHr is 2.84 (95% CI 2.36 to 3.31), mean corpus volume (MCV) is 2.46 (95% CI 1.97 to 2.95), ferritin is 2.37 (95% CI 1.63 to 3.11), and transferrin saturation (TSAT) is 3.76 (95% CI 2.14 to 5.38). To diagnose IDA, the sensitivity value of the CHr concentration was found as 83.5% (95% CI 76.1 to 89.8), specificity value to be 91.8% (95% CI 85.5 to 96.4), and mean cut-off value as 28.2 pg.

**Conclusions:**

The results of our study reveal the findings that CHr is a better biomarker than MCV and ferritin used in determining IDA, and its efficacy is lower than TSAT. It is very important to use it routinely for the pre-diagnosis of IDA, which is very important for public health. The groups in the study are heterogeneous but contain bias. Therefore, meta-analyses of studies with less heterogeneity of CHr are needed.

## Abstract (sr)

**Uvod:**

Anemija usled nedostatka gvožđa (IDA) je najčešća vrsta anemije na svetu i ima mnogo negativnih efekata na kvalitet života. Cilj ove meta-analize je da pokaže da je koncentracija retikulocitnog hemoglobina (CHr) efikasnija od rutinski korišćenih parametara u dijagnozi IDA.

**Metode:**

Sprovedeno je sveobuhvatno i sistematično istraživanje gde su upotrebljene međunarodne baze podataka, uključujući PubMed, Web of Science, Cochrane Library, Science Direct i Google Scholar, koje sadrže sve članke objavljene na temu IDA do 29. decembra 2020. Meta-analiza je obuhvatila sedamnaest članaka.

**Rezultati:**

Analizom su utvrđene vrednosti parametara za veličinu efekta Koenovog d (standardizovana srednja razlika). Shodno tome, CHr je 2,84 (95% CI 2,36 do 3,31), srednja zapremina tela (MCV) je 2,46 (95% CI 1,97 do 2,95), feritin je 2,37 (95% CI 1,63 do 3,11), a zasićenje transferinom (TSAT) je 3,76 (95% CI 2,14 do 5,38). Da bi se dijagnostikovala IDA, utvrđena je vrednost osetljivosti koncentracije CHr na 83,5% (95% CI 76,1 do 89,8), vrednost specifičnosti 91,8% (95% CI 85,5 do 96,4), a srednja granična vrednost je 28,2 pg.

**Zaključak:**

Rezultati naše studije ukazuju da je CHr bolji biomarker od MCV i feritina koji se koriste u određivanju IDA, a njegova efikasnost je niža od TSAT. Veoma je važno da se rutinski koristi za preddijagnozu IDA, što je veoma važno za javno zdravlje. Grupe u studiji su bile heterogene, ali imaju elemente pristrasnosti, pa je potrebno sprovesti meta-analize sa manjom heterogenosti CHr.

## Introduction

Iron is an element that has essential functions for human life. While it is found in the structure of hemoglobin (Hb), which provides oxygen transport in the body, it also ensures the fulfilment of iron-related functions by joining the structure of enzyme systems in some tissues [1]. Iron deficiency anemia (IDA) occurs when the iron intake in humans is less than its excretion, in other words, when a negative iron balance occurs in the body (increased need for iron, absorption disorders, chronic blood loss) as a result of insufficient iron for Hb synthesis in the stores [1] [2]. IDA is the most common type of anemia, and it constitutes the most advanced stage of iron deficiency. The World Health Organization (WHO) describes a hemoglobin value of <130.0 g/L in men, <120.0 g/L in women, and <110.0 g/L in pregnant women as anemia [3] [4]. IDA is more common in women than men due to conditions such as menstruation and pregnancy. Adolescence causes an increase in blood pressure due to rapid growth and development and insufficient iron stores. If this condition cannot be compensated, IDA may occur as a result of insufficient intake. The most important reasons for IDA in postmenopausal women and men are the formation and increase of gastrointestinal system (GIS) bleeding [5] [6] [7]. IDA is characterized by hypochromia and microcytosis in erythrocytes, decreased serum ferritin and serum iron levels, TSAT, and increased total iron-binding capacity [4] [8]. Low serum ferritin level in IDA is essential and should not always be associated with IDA. Again, because it is an acute phase reactant, its normal condition does not exclude IDA; the underlying etiology must be defined and regulated [9]. In contrast to all these conditions, iron overload reduces the efficiency of iron utilization and induces oxidative stress formation [10]. In addition to these, free erythrocyte zinc protoporphyrin (ER-ZPP), soluble transferrin receptor (sTfR), and reticulocyte hemoglobin content (CHr or Ret-He) are among the reliable laboratory test parameters used to describe IDA. Soluble transferrin receptor with increasing erythrocyte ER-ZPP value causes early deterioration of iron condition and emergence of IDA [11] [12] [13] [14].

Bone marrow erythropoietic activity and intracellular iron requirement are important criteria in determining sTfR level. Therefore, in conditions associated with iron deficiency and induced erythropoiesis (sickle cell anemia, megaloblastic anemia, thalas semia, polycythemia, etc.), sTfR concentration increases, while aplastic anemia decreases [15] [16]. Normal serum sTfR level is 3.5-8.5 mg/L. It is known that a high sTfR (>8.5 mg/L) level is an early and sensitive biomarker for the diagnosis of IDA [11].

The ratio of sTfR concentration to logarithmic ferritin level is also determinant in the differential diagnosis of IDA. A ratio of less than 1 is associated with chronic disease anemia, while the ratio higher than 2 is evaluated in favour of IDA [17].

The decrease in iron concentration increases zinc transport in the intestines, and therefore the increased concentration of ER-ZPP (80 μg/dL) in erythrocytes is associated with iron deficiency. However, routine use of ER-ZPP measurements is difficult and time-consuming due to automation difficulties [18].

CHr, also known as Ret-He, measures the amount of hemoglobin in reticulocytes and is an indicator of cell hemoglobination, reflecting the quality of newly produced reticulocytes. Microcytic, hypochromic red blood cell (RBC) is formed due to ongoing reticulocyte production when there is not enough iron. Thus, RET-He reflects an earlier measure of reduced hemoglobin status compared to hemoglobin and hematocrit [11] [19].

Reticulocytes are separated from the erythroblasts after Hb synthesis, pass into the peripheral blood and turn into mature erythrocytes within a few days. Therefore, CHr is the ideal parameter to be considered for real-time Hb synthesis. Reticulocyte hemoglobin content is affected only by the amount of iron unless there are hematopoietic disorders [10].

Determination of iron status is possible with RET-He measurement. RET-He is determined by automated fluorescence flow cytometry, which measures the mean values of the forward light scattering intensity of mature red blood cells and reticulocytes using a polymethine dye. The values obtained reflect the reticulocyte hemoglobin content [20]. Reticulocyte hemoglobin content is more effective in diagnosing iron deficiency, determining early iron deficiency anemia, differentiation of beta-thalassemia feature, and more effective than the other parameters involved in iron metabolism [10] [12]. CHr is a less variable parameter that performs better than ferritin in response to intravenous (IV) iron therapy, providing better diagnostic accuracy for iron [15] [16].

In the United States and Europe, CHr has been accepted as a marker in iron deficiency with a diagnostic threshold of 29 pg. However, there is no reference value agreed on the best value for its sensitivity and specificity [17].

This study examined the effectiveness of CHr in addition to routine parameters in determining IDA.

## Methods

### Literature Search Strategy

Comprehensive systematic research was carried out using international databases, including PubMed, Web of Science, Cochrane Library, Science Direct, and Google Scholar, to determine all studies on CHr as a biomarker of IDA.

Our study included all articles published until December 29, 2020. The words used as search criteria in this study are as follows: »Reticulocyte hemoglobin AND iron deficiency anemia OR iron deficiency«, »reticulocyte hemoglobin« OR »iron deficiency« OR »iron deficiency anemia«.

This systematic review and meta-analysis were carried out under the guidelines for Preferred Reporting Items for Systematic Reviews and Meta-Analyses (PRISMA).

### Selection Criteria

The full texts of the articles related to the study were examined in terms of exclusion and inclusion criteria. Appropriate studies including clinical features of patients diagnosed with IDA and CHr used as a biomarker in the diagnosis of the disease were included in the meta-analysis. 

Prospective/retrospective cohort, case-control, and cross-sectional studies were found suitable for inclusion in our study. 

**Figure 1:**
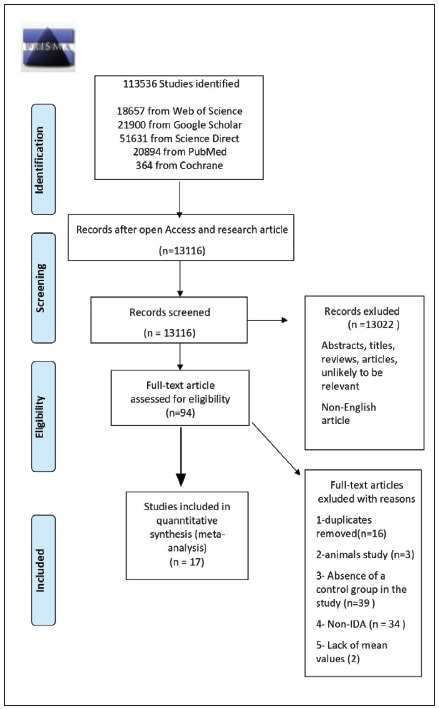
PRISMA flow diagram for inclusion of studies in this meta-analysis

Repeated articles, reviews, case reports, expert opinions, letters, editorials, studies on experimental animals, studies not including control groups, studies where CHr is a biomarker but not related to iron deficiency, studies without available data, studies on results other than mean and standard deviation values, articles published in languages other than English, studies involving chronic patient groups with IDA, and studies only on iron deficiency were excluded (Figure 1).

### Data Extraction and Quality Assessment

Data extraction, evaluation of literature quality, and evaluation of bias risk were carried out independently by two researchers (Serdar M. and Kılıç M.).

The following features were extracted: Article information (first author, year of publication), country, study designs, gender, device information, study example, patient/control information, the total number of samples, researched parameters. This information is shown in Table 1. Microsoft Excel database was used to save the necessary information.

**Table 1:** Features of the studies analyzed for iron deficiency anemia, Sysmex XN Aplpha/10/300/1000, XE 2100/5000 (Sysmex Corporation Kobe, Japan), Technicon H3, (Bayer, Germany), Advia 120, ADVIA 2120i (Siemens AG, Erlangen, Germany) ACD: Anemia of chronic disease, AI: Inflammation anemia, CHr: Reticulocyte hemoglobin content, CKD: Chronic kidney disease, CRF: Chronic renal failure ESRD: End-stage renal disease, ID: Iron deficiency, IDA: Iron deficiency anemia, MCV:Mean corpuscular volume, NIDA: Non-iron deficiency anemia, TM: β-Talasemia minor, TSAT: Transferrin Saturation

FIRST AUTHOR	YEAR	COUNTRY	DEVICE	STUDY DESIGN	PATIENT GROUP	PATIENT/ CONTROL	TOTAL NUMBER	FEMALE	MALE	RESEARCHED PARAMETERS
Chaipokam et al. (46)	2017	Thailand	Sysmex- XE Alpha	Prospective cohort	Adult- Anemia	Control, IDA, Talasemi, Talasemi trait, AI	267	190	77	MCV, CHr
Rehu et al. (35)	2011	Finland	ADVIA 120 and 2120	Retrospective	Adult- Anemia	Control, IDA, ACD	250	138	112	CHr, MCV
Brugnara et al. (8)	1999	Amerika	Technicon H3		Iron Deficiency Children	Control, IDA, ID	210	90	210	CHr, MCV, Ferritin
Balci et al. (47)	2016	Turkey	Sysmex ADVIA 2120i	Case-control	6–12 Years Old Children	Control, IDA, B12 vitamin deficiency, mixed anemia	112	52	60	CHr, MCV, Ferritin
Ceylan et al. (48)	2007	Turkey	ADVIA 120	Case-control	Adult- Anemia	Control, IDA, ID, Talasemi minor, B12 vitamin deficiency	131	91	40	CHr, MCV
Cai et al. (49)	2017	China	ADVIA 120	Case-control	Adults	Control, IDA, NIDA	140	100	40	CHr, MCV, Ferritin
LUO et al. (50)	2007	China	ADVIA 120	Case-control	Pre- menopausal Women- Anemia	Control, IDA, NIDA	142	142		CHr, MCV
Dinh et al. (51)	2020	Vietnam	Sysmex ADVIA 2120i	Retrospective	Adult-ESRD	Control, IDA, NIDA, ESRD, IDA-ESRD	312	188	124	CHr, MCV
Ageeli et al. (52)	2013	Saudi Arabia	ADVIA 2120i	Case-control	Adult- Anemia	Control, IDA, ACD, CRF	320	170	150	CHr, MCV, Ferritin, TSAT
Buttarello et al. (53)	2016	Italy	Sysmex XE-5000	Case-control	Adult- Anemia	Control, IDA, ID, NIDA, trait b talasemia	290			CHr, MCV, Ferritin
Uçar et al. (33)	2019	Turkey	Sysmex XN 1000	Case-control	Adult- Anemia	Control, IDA, ID, NIDA	217	171	46	CHr, MCV, Ferritin,TSAT
Toki et al. (54)	2017	Japan	Sysmex XN 300/XE	Case-control	Adult- Anemia	Control, IDA, ID, NIDA	211	148	63	CHr, MCV, Ferritin,TSAT
Vázquez-López et al. (55)	2019	Spain	ADVIA 120	Case-control	1–16 Years Old Children	Contol, IDA, ID	1239	620	619	CHr, MCV, Ferritin,TSAT
Malczewska- Lenczowska et al. (56)	2017	Poland	ADVIA 120	Case-control	Sports women	Control, ID stage I, ID stage II	219	219		CHr, MCV, Ferritin
Chinudomwong et al. (57)	2020	Thailand	Sysmex XN-10	Case-control	Adult- Anemia	Control, IDA, IDA-inflamma- tion, NIDA-AI, NIDA-CKD, Talasemi	938	603	335	CHr, MCV, Ferritin
Urrechaga et al. (58)	2011	Spain	Sysmex XE 5000	Case-control	Anemia and Talasemia	Control, talasemia trait, mild IDA, severe IDA	473			CHr, MCV, Ferritin, TSAT
Rungngu et al. (59)	2016	Indonesia	Sysmex XE-2100	Cross-section- al	6–12 Years Old Children- Anemia	Control, IDA, NIDA	50	17	33	CHr, Ferritin

### Statistical Analysis

The meta-analysis study was performed using MedCalc statistical software, version 19.0.7 (Med-Calc Software, Ostend, Belgium). A meta-analysis was performed on Cohen's d effect size (Standardized Mean Difference) by taking the mean and standard deviation values. In the evaluation, the results obtained with the random-effects model of the studies with low bias risk were taken into consideration. The study aimed to perform group analysis of CHr, MCV, ferritin, and TSAT biomarkers according to the target range in IDA. Using Cochran's Q statistic and I^2^ statistic respectively, statistical heterogeneity was evaluated. If the I^2^ statistical value was above 50 per cent and the p-value was lower than 0.05, the heterogeneity was considered statistically significant.

### Publication Bias

Two reviewers evaluated independently the risk of bias in each study using the Diagnostic Precision Study Quality Assessment Tool (QUADAS-2) recommended [18]. Publication bias is demonstrated by analysis with a funnel plot.

## Results

### Literature Search and Study Characteristics

Based on the search strategy, 113,536 studies were evaluated among the databases specified. A total of 13,116 studies were included in the study after the records, except for studies with open access and research articles were excluded. Thirteen thousand twenty-two articles not suitable for the title and abstracts and published in languages other than English were excluded. From the remaining 94 pieces, 17 of them were included in the meta-analysis, excluding those with repeating records, animal studies, those that did not include a control group, and those related to chronic diseases other than iron deficiency. The flowchart and detailed literature search steps are shown in Figure 1.

### Characteristics and Quality Studies

The articles on CHr, a biomarker used in patients with IDA, were included in our study, all in English until December 29, 2020. From the 17 articles, 2 of them were written in Thailand, 3 in Turkey, 2 in Spain, 1 in Finland, 1 in the United States, 2 in China, and one in Indonesia, Vietnam, Saudi Arabia, Italy, Poland, and Japan. The study contains 951 individuals with IDA and 3491 people, including 2540 control group members. The features of the included studies are shown in Figure 1.

### Reticulocyte Hemoglobin Content

**Figure 2:**
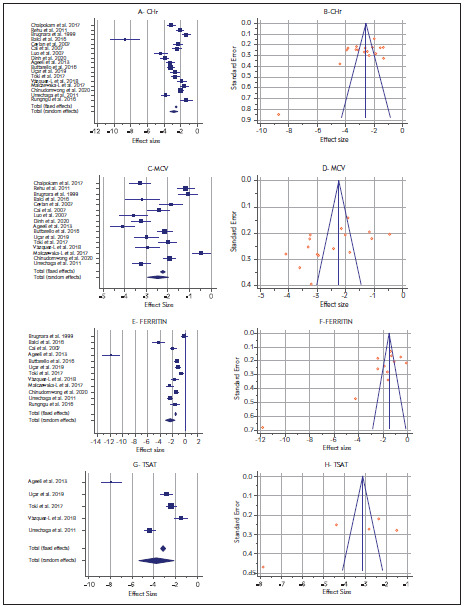
A Forest plot of reticulocyte hemoglobin content (CHr)<br>B Funnel plot of reticulocyte hemoglobin content<br>C Forest plot of mean corpuscular volume (MCV)<br>D Funnel plot of mean corpuscular volume<br>E Forest plot of ferritin<br>F Funnel plot of ferritin<br>G Forest plot of transferrin saturation (TSAT)<br>H Funnel plot of transferrin saturation

As a result of a detailed literature review, 17 articles including IDA data of CHr were included. In the meta-analysis performed with the low bias risk random-effects model in the included articles, the I^2^ value of CHr was 93.52%, so intergroup heterogeneity was achieved (P <0.0001), and random effect size value 2.84 (95% CI 2.36 to 3.31) is shown in Table 2. Publication bias is evaluated with a forest plot in Figure 2A and funnel plot in Figure 2B.

**Table 2:** Meta-analysis of reticulocyte hemoglobin content (CHr), Mean Corpuscular Volume (MCV), ferritin, Transferrin Saturation (TSAT), sensitivity and specificity of CHr to diagnose iron deficiency anemia articles CI = Confidence interval, n = Sample size, SD = Standard Deviation

Study	IDA (n)	IDA-CHr Main±SD	Control (n)	Control- CHr Main±SD	Effect Size	95%<br> CI	MCV		FERRITIN		TSAT		SENSITIVITY			SPECIFICITY		
							Effect Size	95%<br> CI	Effect Size	95%<br> CI	Effect Size	95%<br> CI	Sample size	Proportion (%)	95%<br> CI	Sample size	Proportion (%)	95%<br> CI
Chaipokam et al. 2017 (46)	53	21.2±5.5	99	33.1±2.4	-3.137	-3.62 to -2.65	-3.30	-3.80 to - 2.80					53	83.0	70.2 to 91.9	99	80.8	71.6 to 88.0
Rehu et al. 2011 (35)	58	26.8±3.8	63	33.2±2	-2.12	-2.57 to -1.67	-1.16	-1.54 to - 0.77					58	82.7	70.5 to 91.4	63	90.4	80.4 to 96.4
Brugnara et al. 1999 (8)	24	24.2±2.7	186	26.8±1.8	-1.349	-1.79 to -0.90	-1.08	-1.51 to - 0.64	-0.09	-0.52 to 0.33			24	79.1	57.8 to 92.8	186	74.7	67.8 to 80.8
Balci et al. 2016 (47)	26	22.26±1	32	29.9±0.7	-8.692	-10.39 to -6.99	-3.20	-3.99 to - 2.41	-4.26	-5.21 to -3.31								
Ceylan et al. 2007 (48)	41	21.8±3.3	34	28.2±1.7	-2.349	-2.94 to -1.75	-1.85	-2.40 to - 1.30					41	85.3	70.8 to 94.43	34	1	84.6 to 99.9
Cai et al. 2007 (49)	56	23.3±4	46	31.8±2.5	-2.476	-2.99 to -1.95	-2.42	-2.93 to - 1.90	-1.91	-2.38 to -1.44			56	87.5	75.9 to 94.82	46	91.3	79.2 to 97.5
Luo et al. 2007 (50)	30	23.5±3.1	71	32±1.1	-4.403	-5.15 to -3.65	-3.609	-4.27 to - 2.94										
Dinh et al. 2020 (51)	59	23.4±3.2	145	31.2±1.2	-3.90	-4.38 to -3.41	-3.256	-3.69 to - 2.81					59	98.3	90.9 to 99.9	145	97.9	94.0 to 99.5
Ageeli et al. 2013 (52)	100	22.9±2.9	60	30.9±1.3	-3.27	-3.76 to -2.79	-4.093	-4.64 to - 3.53	-11.85	-13.2 to -10.5	-7.89	-8.82 to -6.96						
Buttarello et al. 2016 (53)	58	24.4±4.8	164	33±1.2	-3.23	-3.66 to -2.80	-2.141	-2.50 to - 1.78	-1.31	-1.63 to -0.99			58	91.3	81.0 to 97.1	164	94.5	89.8 to 97.4
Uçar et al. 2019 (33)	52	21±4.1	54	36.6±7	-2.69	-3.22 to -2.15	-2.996	-3.55 to - 2.43	-1.10	-1.51 to -0.69	-2.83	-3.37 to -2.29	52	90.3	78.9 to 96.8	54	100	93.3 to 100.0
Toki et al. 2017 (54)	72	23.4±4.9	67	33.8±2.5	-2.631	-3.08 to -2.17	-2.008	-2.41 to - 1.59	-0.56	-0.90 to -0.22	-2.38	-2.82 to -1.94						
Vázquez-L et al. 2019 (55)	13	24.5±30	1153	31.1±1.7	-1.885	-2.43 to -1.33	-2.951	-3.51 to - 2.39	-1.64	-2.19 to -1.09	-1.51	-2.06 to -0.96						
Malczewska-L et al. 2017 (56)	33	29.3±1.8	87	31.4±1.0	-1.567	-2.02 to -1.12	-0.457	-0.86 to -0.05	-2.44	-2.94 to -1.93								
Chinudomwong et al. 2020 (57)	133	20.6±9	155	33±1.4	-1.993	-2.27 to -1.71	-1.909	-2.18 to -1.63	-1.40	-1.66 to -1.14			133	73.6	65.3 to 80.9	155	96.7	92.6 to 98.9
Urrechaga et al. 2011 (58)	126	22.3±3.7	90	33.7±1.4	-3.827	-4.28 to -3.37	-3.235	-3.64 to -2.82	-2.44	-2.80 to -2.08	-4.39	-4.89 to -3.89						
Rungngu et al. 2016 (59)	16	25.8±4.8	34	29.8±1.3	-1.338	-1.99 to -0.68			-1.59	-2.27 to -0.91			16	43.7	19.7 to 70.1	34	85.2	68.9 to 95.0
Total (random effects)	950		2540		-2.846	-3.31 to -2.38	-2.463	-2.95 to -1.97	-2.38	-3.11 to -1.63	-3.77	-5.38 to -2.15	550	83.5	76.1 to 89.9	980	91.8	85.6 to 96.4

### Mean Corpuscular Volume

After the literature review, 16 articles with MCV data were included. In the meta-analysis performed with the low bias risk random-effects model in the included articles, the MCV I^2^ value was 94.71%, so intergroup heterogeneity was achieved (P <0.0001), and random effect size value 2.46 (95% CI 1.97 to 2.95) is shown in Table 2. Publication bias is evaluated with a forest plot Figure 2C and funnel plot in Figure 2D. 

### Ferritin

12 articles with ferritin data were included. In the meta-analysis performed with the low bias risk random-effects model in the included articles, the ferritin I^2^ value was 97.15%, so intergroup heterogeneity was achieved (P<0.0001), and random effect size value 2.37 (95% CI 1.63 to 3.11) is shown in Table 2. Publication bias is evaluated with a forest plot Figure 2E and funnel plot in Figure 2F.

### Transferrin Saturation

5 articles with TSAT data were included. In the meta-analysis performed with the low bias risk model of random effects in the included articles, the TSAT I^2^ value was 97.70%. Therefore, intergroup heterogeneity was achieved (P<0.0001), and random effect size value 3.76 (95% CI 2.14 to 5.38) is shown in Table 2. Publication bias is evaluated with a forest plot Figure 2G and funnel plot in Figure 2H. 

## Discussion

Anemia is a global health problem that is quite common worldwide and affects 43% of children under the age of five, 38% of pregnant women, and 29% of non-pregnant women [19]. IDA brings along many complications such as growth retardation, neurocognitive deficiencies, impaired immune system, increased risk of premature, and impaired learning ability [20] [21] [22] [23] [24]. It is therefore important to ensure accurate and timely diagnosis of the disease by preventing such adverse effects [25] [26] [27]. Although there is no single and best test for determining iron deficiency, bone marrow aspiration, which is accepted as the gold standard, and the method of staining bone marrow macrophages and erythroid precursors with Prussian blue is used. However, the method is not suitable for routine use because it is expensive, subjective, and invasive [26] [28].

There are many biochemical parameters used in the diagnosis of IDA. However, as these parameters are affected by certain conditions, it is not easy to evaluate them. Serum ferritin concentration, serum iron level, TSAT, and total iron-binding capacity (TIBC) are the most widely used biochemical tests. Although serum ferritin level reveals the iron concentration accumulated in the body, factors such as acute and chronic inflation, malignancy, liver diseases, and excessive alcohol use increase independent of iron [29]. Serum iron level decreases with infection, inflammation, and malignancy but increases with liver disease. Since the TSAT level is calculated on iron and TIBC, it is affected by changes in these values and does not always give an accurate result [29] [30] [31] [32].

In recent years, CHr has become one of the parameters used to determine IDA [33] [34]. reticulocytes, as the first erythrocytes produced in the bone marrow, transform into mature red blood cells a day or two after entering the bloodstream. Reticulocyte parameters have become one of the parameters used to reflect the iron status in a short time due to their shorter lifespan compared to erythrocytes and ability to provide information about bone marrow erythrocyte production [12] [35]. CHr has a higher specificity and a lower coefficient of variation since it is not affected by inflammation like some parameters used in the diagnosis of IDA [36]. Reticulocyte hemoglobin content data can be obtained from a few millimeters of peripheral blood compared to bone marrow biopsy and is also advantageous because it is relatively inexpensive, convenient, and less invasive [37].

In this random-effect meta-analysis, studies on the efficiency of CHr in the diagnosis of IDA are summarized. For this meta-analysis, a total of 13,116 articles were examined, and 17 studies were included in the study according to the inclusion and exclusion criteria. The number of articles including parameters such as MCV, ferritin, and TSAT, which were used frequently in the past in the diagnosis of iron deficiency, varies (16 articles for MCV values, 12 articles for Ferritin values, and 5 articles for TSAT values were examined). The most effective examination is thought to be obtained by including each study that meets the criteria given in Figure 1 for meta-analysis. The literature review exhibited the fact that this study is the first meta-analysis of CHr to determine the diagnosis of IDA.

A total of 3491 individuals, of which 2540 people were in the control group, and 951 people were with IDA, were included in this study. The inclusion criteria for the current study were determined as CHr, which is one of the important parameters in the diagnosis of IDA and containing at least one of the parameters of MCV, ferritin, or TSAT.

Ferritin is an indicator of the total amount of iron stored in the body. When the serum ferritin level shows values less than 15 mg/L, it is highly suggestive for the diagnosis of IDA. At values below 30 mg/L, the sensitivity is 92%, and the specificity is 98% [38] [39]. Again, a ferritin level below 45 mg/L and the sensitivity of 85% and specificity of 92% are particular to IDA [40] [41].

A low MCV value alone is not sufficient for a diagnosis of IDA. Unless the MCV volume is greater than 95 μm] (95 fL), it should not be considered in IDA because this threshold has a sensitivity of 97.6% [40] [42].

TSAT is low in IDA, typically less than 10%, and in this case, the sensitivity is 48%, and the specificity is 88% [43] [44].

**Figure 3:**
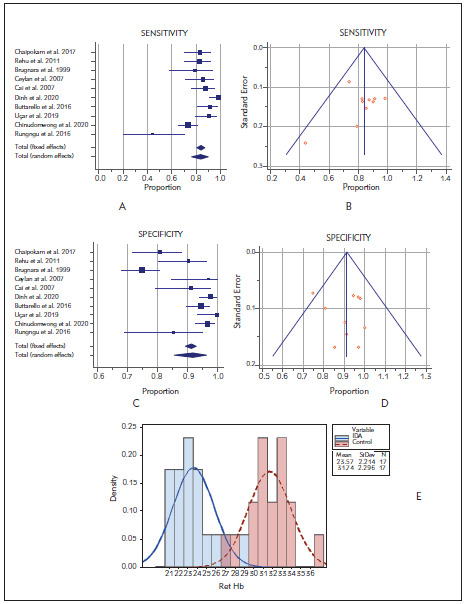
A Forest plot meta-analysis of the sensitivity of reticulocyte hemoglobin content to diagnose iron-deficiency anemia articles<br>B Funnel plot meta-analysis of the sensitivity of reticulocyte hemoglobin content to diagnose iron-deficiency anemia articles<br>C Forest plot meta-analysis of the specificity of reticulocyte hemoglobin content to diagnose iron-deficiency anemia articles<br>D Funnel plot meta-analysis of the specificity of reticulocyte hemoglobin content to diagnose iron-deficiency anemia articles, the reticulo cyte hemoglobin content mean cut-off value

In the meta-analysis performed with the low bias risk random-effects model in the included articles, the sensitivity CHr I^2^ value was 78.78% (95% CI 61.43 to 88.32), so intergroup heterogeneity was achieved (P<0.0001) (Figure 3A–B). The sensitivity of CHr to diagnose IDA was found as 83.5% (95% CI 76.1 to 89.8), and they are shown in Table 2. 

In the meta-analysis performed with the low bias risk random-effects model in the included articles, the specificity CHr I^2^ value was 89.53% (95% CI 82.88 to 93.60), so intergroup heterogeneity was achieved (P<0.0001) (Figure 3C–D). The sensitivity of CHr to diagnose IDA was found as 91.8% (95% CI 85.5 to 96.4), and they are shown in Table 2. 

In conclusion, the meta-analysis study showed that CHr is a better marker than other more commonly used parameters in IDA. Many previous studies also support this conclusion. Also, this meta-analysis we conducted is important for being the first meta-analysis study regarding CHr and IDA.

The literature review revealed that different cutoff results related to CHr were obtained, and these values vary between 28-29 pg. The CHr mean cutoff value obtained in our study is 28.2, and this is shown in the histogram in Figure 3E.

**Figure 4:**
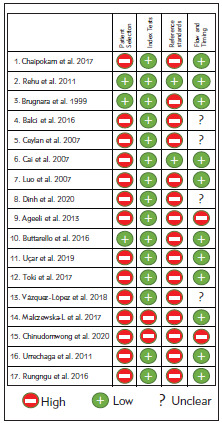
Methodological quality of the included studies<br>(individual assessment)

There are important heterogeneity and bias problems in the studies. Patient selection and lack of reference methods are particularly important. There are important criteria differences for index tests. Also, it is important to have significant group differences (pediatric patients, renal failure, etc.) in studies. It should be noted that there will be a significant change in methodologies over time (Figure 4).

All parameters of IDA, which are included in the research, have heterogeneity. CHr is a more effective marker in determining IDA compared to the routinely used MCV and ferritin levels. The effect size value of TSAT, one of the parameters examined, is above 0.80, and its selectivity is higher than CHr.

The study also has some limitations. These are as follows: like other meta-analysis studies in the literature, methodological differences are arising from combining studies conducted with different methods, and this may lead to bias. Since studies in which the diagnosis of IDA of CHr was evaluated in the metaanalysis were included, many parameters used routinely were excluded. IDA, erythropoiesis status, and chronic anemias were excluded from the study. In the study, no distinction was made according to gender and age.

## Conclusion

This study is the first meta-analysis to evaluate the efficiency of CHr in the diagnosis of IDA. According to our results, CHr should be used additionally with the parameters used in the diagnosis of IDA. 'The results of our study reveal the findings that CHr is a better biomarker than MCV and ferritin used in determining IDA, and its efficacy is lower than TSAT. It is very important to routinely use it for the pre-diagnosis of IDA, which is very important for public health.' CHr alone provides important information about the current bioavailability of iron, but its use with other parameters removes uncertainty about the diagnosis and treatment of IDA. CHr is a very important parameter that can be used to evaluate a very common disease in the clinic, such as IDA' The heterogeneity index of the study results is quite high. Therefore, comprehensive studies with more homogeneous groups are needed to elucidate the relationship between IDA and CHr.

## Dodatak

### Acknowledgements

Not applicable.

### Conflict of interest statement

All the authors declare that they have no conflict of interest in this work.

## References

[1] Baker R D, Greer F R (2010). Diagnosis and Prevention of Iron Deficiency and Iron-Deficiency Anemia in Infants and Young Children (0-3 Years of Age). Pediatrics.

[2] Aslan Y, Erduran E, Mocan H, Gedik Y, Okten A, Soylu H, Değer O (1997). Absorption of iron from grape-molasses and ferrous sulfate: A comparative study in normal subjects and subjects with iron deficiency anemia. Turk J Pediatr.

[3] Guralnik J M, Eisenstaedt R S, Ferrucci L, Klein H G, Woodman R C (2004). Prevalence of anemia in persons 65 years and older in the United States: Evidence for a high rate of unexplained anemia. Blood.

[4] Joosten E (2018). Iron deficiency anemia in older adults: A review. Geriatr Gerontol Int.

[5] Agha F, Sadaruddin A, Khan R A, Ghafoor A (1992). Iron deficiency in adolescents. J Pak Med Assoc.

[6] McMahon L P (2010). Iron deficiency in pregnancy. Obstet Med.

[7] Kim B S M (2014). Diagnosis of gastrointestinal bleeding: A practical guide for clinicians. World J Gastrointest Pathophysiol.

[8] Brugnara C (1999). Reticulocyte hemoglobin content to diagnose iron deficiency in children. JAMA.

[9] Powers J M, Buchanan G R, Adix L, Zhang S, Gao A, McCavit T L (2017). Effect of Low-Dose Ferrous Sulfate vs Iron Polysaccharide Complex on Hemoglobin Concentration in Young Children With Nutritional Iron-Deficiency Anemia. JAMA.

[10] Ogawa C, Tsuchiya K, Maeda K (2020). Reticulocyte hemoglobin content. Clin Chim Acta.

[11] Mast A E, Blinder M A, Dietzen D J (2008). Reticulocyte hemoglobin content. Am J Hematol.

[12] Ullrich C (2005). Screening Healthy Infants for Iron Deficiency Using Reticulocyte Hemoglobin Content. JAMA.

[13] Buttarello M, Pajola R, Novello E, Rebeschini M, Cantaro S, Oliosi F, Naso A, Plebani M (2010). Diagnosis of Iron Deficiency in Patients Undergoing Hemodialysis. Am J Clin Pathol.

[14] Chuang C - L (2003). Early prediction of response to intravenous iron supplementation by reticulocyte haemoglobin content and high-fluorescence reticulocyte count in haemodialysis patients. Nephrol Dial Transplant.

[15] Gaweda A E (2017). Markers of iron status in chronic kidney disease. Hemodial Int.

[16] Mittman N, Sreedhara R, Mushnick R, Chattopadhyay J, Zelmanovic D, Vaseghi M, Avram M M (1997). Reticulocyte hemoglobin content predicts functional iron deficiency in hemodialysis patients receiving rHuEPO. Am J Kidney Dis.

[17] Majoni S W, Lawton P D, Rathnayake G, Barzi F, Hughes J T, Cass A (2020). Narrative Review of Hyperferritinemia, Iron Deficiency, and the Challenges of Managing Anemia in Aboriginal and Torres Strait Islander Australians With CKD. Kidney Int Rep.

[18] Whiting P F (2011). QUADAS-2: A Revised Tool for the Quality Assessment of Diagnostic Accuracy Studies. Ann Intern Med.

[19] Hastka J, Lasserre J J, Schwarzbeck A, Hehlmann R (1994). Central role of zinc protoporphyrin in staging iron deficiency. Clin Chem.

[20] Pasricha S - R, Drakesmith H (2016). Iron Deficiency Anemia. Hematol Oncol Clin North Am.

[21] Kiudelienė R, Griniūtė R, Labanauskas L (2008). Prognostic value of reticulocyte hemoglobin content to diagnose iron deficiency in 6-24-month-old children. Medicina (B Aires).

[22] Hempel E V, Bollard E R (2016). The Evidence-Based Evaluation of Iron Deficiency Anemia. Med Clin North Am.

[23] Turgeon O'Brien H, Blanchet R, Gagné D, Lauzière J, Vézina C (2016;). Using Soluble Transferrin Receptor and Taking Inflammation into Account When Defining Serum Ferritin Cutoffs Improved the Diagnosis of Iron Deficiency in a Group of Canadian Preschool Inuit Children from Nunavik. Anemia.

[24] Kassebaum N J (2016). The Global Burden of Anemia. Hematol Oncol Clin North Am.

[25] Ennis K M, Dahl L V, Rao R B, Georgieff M K (2018). Reticulocyte hemoglobin content as an early predictive biomarker of brain iron deficiency. Pediatr Res.

[26] Lopez A, Cacoub P, Macdougall I C, Peyrin-Biroulet L (2016). Iron deficiency anaemia. Lancet.

[27] Mehta S, Goyal L K, Kaushik D, Gulati S, Sharma N, Harshvardhan L, et al (2016). Reticulocyte Hemoglobin vis-avis Serum Ferritin as a Marker of Bone Marrow Iron Store in Iron Deficiency Anemia. J Assoc Physicians India.

[28] Braga F, Infusino I, Dolci A, Panteghini M (2014). Soluble transferrin receptor in complicated anemia. Clin Chim Acta.

[29] Jimenez K, Kulnigg-Dabsch S, Gasche C (2015). Management of Iron Deficiency Anemia. Gastroenterol Hepatol (N Y).

[30] Fishbane S, Galgano C, Langley R C, Canfield W, Maesaka J K (1997). Reticulocyte hemoglobin content in the evaluation of iron status of hemodialysis patients. Kidney Int.

[31] Karagülle M, Gündüz E, Mutlu F Ş, Akay M O (2013). Clinical Significance of Reticulocyte Hemoglobin Content in the Diagnosis of Iron Deficiency Anemia. Turk J Haematol.

[32] Duman E, Kulaksizoglu S, Cifc E, Ozulku M (2017). Is there a real correlation between red cell distribution width and peripheral arterial disease?. J Med Biochem.

[33] Uçar M A, Falay M, Dagdas S, Ceran F, Urlu S M, Özet G (2019). The importance of RET-He in the diagnosis of iron deficiency and iron deficiency anemia and the evaluation of response to oral iron therapy. J Med Biochem.

[34] Bahrainwala J, Berns J S (2016). Diagnosis of Iron-Deficiency Anemia in Chronic Kidney Disease. Semin Nephrol.

[35] Rehu M, Ahonen S, Punnonen K (2011). The diagnostic accuracy of the percentage of hypochromic red blood cells (%HYPOm) and cellular hemoglobin in reticulocytes (CHr) in differentiating iron deficiency anemia and anemia of chronic diseases. Clin Chim Acta.

[36] Kotisaari S, Romppanen J, Penttilä I, Punnonen K (2002). The Advia 120 red blood cell and reticulocyte indices are useful in diagnosis of iron-deficiency anemia. Eur J Haematol.

[37] Fishbane S, Shapiro W, Dutka P, Valenzuela O F, Faubert J (2001). A randomized trial of iron deficiency testing strategies in hemodialysis patients. Kidney Int.

[38] Ermens A A M, Hoffmann J J M L, Krockenberger M, Van Wijk E M (2012). New erythrocyte and reticulocyte parameters on CELL-DYN Sapphire: Analytical and preanalytical aspects. Int J Lab Hematol.

[39] Camaschella C (2015). Iron-Deficiency Anemia. Longo DL, editor. N Engl J Med.

[40] Mast A E, Blinder M A, Gronowski A M, Chumley C, Scott M G (1998). Clinical utility of the soluble transferrin receptor and comparison with serum ferritin in several populations. Clin Chem.

[41] Ioannou G N, Spector J, Scott K, Rockey D C (2002). Prospective evaluation of a clinical guideline for the diagnosis and management of iron deficiency anemia. Am J Med.

[42] Guyatt G H, Patterson C, Ali M, Levine M, Turpie I, Meyer R, Singer J (1990). Diagnosis of iron-deficiency anemia in the elderly. Am J Med.

[43] Bermejo F, García-López S (2009). A guide to diagnosis of iron deficiency and iron deficiency anemia in digestive diseases. World J Gastroenterol.

[44] Punnonen K, Irjala K, Rajamäki A (1997). Serum Transferrin Receptor and Its Ratio to Serum Ferritin in the Diagnosis of Iron Deficiency. Blood.

[45] Guyatt G H, Oxman A D, Ali M, Willan A, McIlroy W, Patterson C (1992). Laboratory diagnosis of iron-deficiency anemia: An overview. J Gen Intern Med.

[46] Chaipokam J, Na N T, Ponlapat R (2016). Diagnostic accuracy of reticulocyte hemoglobin content in Thai patients with microcytic red cells as a test for iron deficiency anemia. Asian Biomed.

[47] Balci Y, Akpinar F, Polat A, Uzun U, Ergin A (2016). Evaluation of Reticulocyte Parameters in Iron Deficiency, Vitamin B12 Deficiency and Mixed Anemia. Clin Lab.

[48] Ceylan C, Miskioğlu M, Çolak H, Kiliççioğlu B, Özdemir E (2007). Evaluation of reticulocyte parameters in iron deficiency, vitamin B(12) deficiency and beta-thalassemia minor patients. Int J Lab Hematol.

[49] Cai J, Wu M, Ren J, Du Y, Long Z, Li G, Han B, Yang L (2017). Evaluation of the Efficiency of the Reticulocyte Hemoglobin Content on Diagnosis for Iron Deficiency Anemia in Chinese Adults. Nutrition.

[50] Luo D, Chen Y, Wu W, Zhang F, Xu J, Cui W, Li S, Li R (2007). Reticulocyte hemoglobin content in the diagnosis of iron deficiency in Chinese pre-menopausal women. Chin Med J (Engl).

[51] Dinh N H, Cheanh B S M, Tran L T A (2020). The validity of reticulocyte hemoglobin content and percentage of hypochromic red blood cells for screening iron-deficiency anemia among patients with end-stage renal disease: A retrospective analysis. BMC Nephrol.

[52] Ageeli A A, Algahtani F H, Alsaeed A H (2013). Reticulocyte Hemoglobin Content and Iron Deficiency: A Retrospective Study in Adults. Genet Test Mol Biomarkers.

[53] Buttarello M, Pajola R, Novello E, Mezzapelle G, Plebani M (2016). Evaluation of the hypochromic erythrocyte and reticulocyte hemoglobin content provided by the Sysmex XE-5000 analyzer in diagnosis of iron deficiency erythropoiesis. Clin Chem Lab Med.

[54] Toki Y, Ikuta K, Kawahara Y, Niizeki N, Kon M, Enomoto M, Tada Y, Hatayama M, Yamamoto M, Ito S, Shindo M, Kikuchi Y, Inoue M, Sato K, Fujiya M, Okumura T (2017). Reticulocyte hemoglobin equivalent as a potential marker for diagnosis of iron deficiency. Int J Hematol.

[55] Vázquez-López M A, López-Ruzafa E, Ibáñez-Alcalde M, Martín-González M, Bonillo-Perales A, Lendínez-Molinos F (2019). The usefulness of reticulocyte haemoglobin content, serum transferrin receptor and the sTfR-ferritin index to identify iron deficiency in healthy children aged 1-16 years. Eur J Pediatr.

[56] Malczewska-Lenczowska J, Orysiak J, Szczepańska B, Turowski D, Burkhard-Jagodzińska K, Gajewski J (2017). Reticulocyte and erythrocyte hypochromia markers in detection of iron deficiency in adolescent female athletes. Biol Sport.

[57] Chinudomwong P, Binyasing A, Trongsakul R, Paisooksantivatana K (2020). Diagnostic performance of reticulocyte hemoglobin equivalent in assessing the iron status. J Clin Lab Anal.

[58] Urrechaga E, Borque L, Escanero J F (2011). Erythrocyte and reticulocyte parameters in iron deficiency and thalassemia. J Clin Lab Anal.

[59] Rungngu S L P, Wahani A, Mantik M F J (2016). Reticulocyte hemoglobin equivalent for diagnosing iron deficiency anemia in children. Paediatr Indones.

